# Diaqua­tris­(nitrato-κ^2^ *O*,*O*′){2,2′-[pyridine-2,6-diylbis(methyl­ene­oxy)]dibenzaldehyde-κ*O* ^1^}dysprosium(III)–2,2′-[pyridine-2,6-diylbis(methyl­ene­oxy)]dibenzaldehyde (1/1)

**DOI:** 10.1107/S160053681203680X

**Published:** 2012-09-05

**Authors:** Sara Luisa Rodríguez de Luna, Perla Elizondo, Sylvain Bernès, Marcos Flores-Alamo, Leyda E. López

**Affiliations:** a Universidad Autónoma de Nuevo León, UANL, Facultad de Ciencias Químicas, Av. Universidad S/N, Ciudad Universitaria, San Nicolás de los Garza, Nuevo León CP 66451, Mexico; b Facultad de Química, Universidad Nacional Autónoma de México, México, D.F. 04510, Mexico

## Abstract

The title compound, [Dy(NO_3_)_3_(C_21_H_17_NO_4_)(H_2_O)_2_]·C_21_H_17_NO_4_, may be considered as an organic–metalorganic 1:1 co-crystal, in which the two dialdehyde mol­ecules act as a ligand and as an organic moiety, respectively. The Dy^III^ atom coordinates nine O atoms from the organic ligand, bidentate nitrate ions and water mol­ecules, approximating a square-face-tricapped trigonal–prismatic geometry. The coordinated dialdehyde is not planar: the uncoordinated oxybenzaldehyde group is twisted by 39.96 (4)° from the rest of the ligand. In contrast, the free organic moiety is almost planar, with an r.m.s. deviation of 0.15 Å. In the crystal, segregated stacks of dialdehyde are formed in the [100] direction. For the complex, the shortest π–π contact is found at 3.781 (2) Å, and for the free ligand, at 3.785 (2) Å. The crystal structure is further stabilized by O—H⋯O and O—H⋯N hydrogen bonds in which coordinated water mol­ecules are the donor groups.

## Related literature

For the X-ray structure of the free ligand and other rare-earth complexes based on this ligand, see: Rodríguez De Luna *et al.* (2010 ▶). For isotypic complexes, see: Garza Rodríguez (2010 ▶). For the nomenclature of 9-coordinated metal centers, see: IUPAC (2005 ▶).
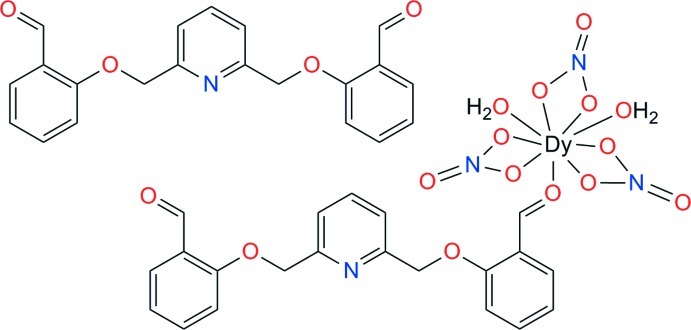


## Experimental

### 

#### Crystal data

[Dy(NO_3_)_3_(C_21_H_17_NO_4_)(H_2_O)_2_]·C_21_H_17_NO_4_
*M* *_r_* = 1079.27
Triclinic, 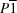

*a* = 7.7552 (3) Å
*b* = 16.1249 (8) Å
*c* = 17.7178 (7) Å
α = 75.531 (4)°
β = 85.173 (3)°
γ = 88.398 (4)°
*V* = 2137.71 (16) Å^3^
*Z* = 2
Mo *K*α radiation
μ = 1.84 mm^−1^
*T* = 136 K
0.43 × 0.26 × 0.12 mm

#### Data collection

Agilent Xcalibur Atlas Gemini diffractometer
Absorption correction: analytical [*CrysAlis PRO* (Agilent, 2010 ▶); based on expressions derived by Clark & Reid (1995 ▶)] *T*_min_ = 0.599, *T*_max_ = 0.816
15907 measured reflections
8436 independent reflections
7464 reflections with *I* > 2σ(*I*)
*R*_int_ = 0.034

#### Refinement

*R*[*F* ^2^ > 2σ(*F* ^2^)] = 0.030
*wR*(*F* ^2^) = 0.066
*S* = 1.05
8436 reflections
616 parameters
H atoms treated by a mixture of independent and constrained refinement
Δρ_max_ = 0.95 e Å^−3^
Δρ_min_ = −0.88 e Å^−3^

### 

Data collection: *CrysAlis CCD* (Agilent, 2010 ▶); cell refinement: *CrysAlis CCD* (Agilent, 2010 ▶); data reduction: *CrysAlis RED*; program(s) used to solve structure: *SHELXS97* (Sheldrick, 2008 ▶); program(s) used to refine structure: *SHELXL97* (Sheldrick, 2008 ▶); molecular graphics: *SHELXTL* (Sheldrick, 2008 ▶) and *Mercury* (Macrae *et al.*, 2008 ▶); software used to prepare material for publication: *SHELXTL*.

## Supplementary Material

Crystal structure: contains datablock(s) I, global. DOI: 10.1107/S160053681203680X/vn2050sup1.cif

Structure factors: contains datablock(s) I. DOI: 10.1107/S160053681203680X/vn2050Isup2.hkl

Additional supplementary materials:  crystallographic information; 3D view; checkCIF report

## Figures and Tables

**Table 1:** Selected bond lengths (Å)

Dy1—O1	2.435 (2)
Dy1—O5	2.327 (2)
Dy1—O6	2.320 (2)
Dy1—O7	2.410 (2)
Dy1—O8	2.437 (2)
Dy1—O10	2.443 (2)
Dy1—O11	2.429 (2)
Dy1—O13	2.460 (2)
Dy1—O14	2.403 (2)

**Table 2:** Hydrogen-bond geometry (Å, °)

*D*—H⋯*A*	*D*—H	H⋯*A*	*D*⋯*A*	*D*—H⋯*A*
O5—H51⋯N1^i^	0.76 (4)	1.97 (4)	2.724 (3)	173 (4)
O5—H52⋯O12^ii^	0.73 (3)	2.19 (4)	2.907 (3)	168 (4)
O6—H61⋯O4^iii^	0.71 (3)	2.10 (3)	2.797 (3)	169 (4)
O6—H62⋯N51^iii^	0.86 (3)	1.86 (4)	2.712 (3)	177 (3)

Symmetry codes: (i) 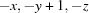; (ii) 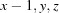; (iii) 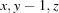.

**Fig. 1.**
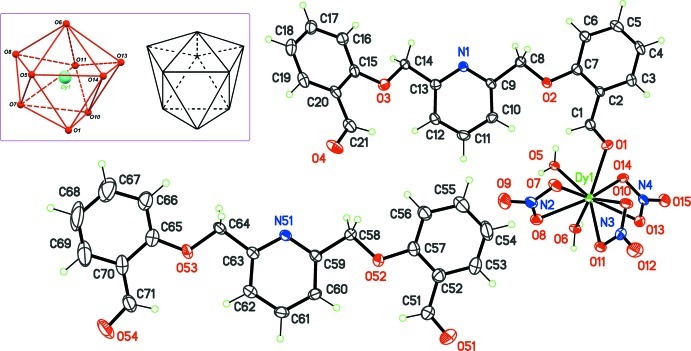
ORTEP-like view of the asymmetric unit, with displacement ellipsoids for non-H atoms at the 50% probability level. The inset represents the TPRS-9 polyhedron formed by coordinated O atoms. On the left, the actual coordination is represented, which compares well with the ideal D3 h polyhedron on the right, depicted in the IUPAC red book (IUPAC, 2005).

**Fig. 2.**
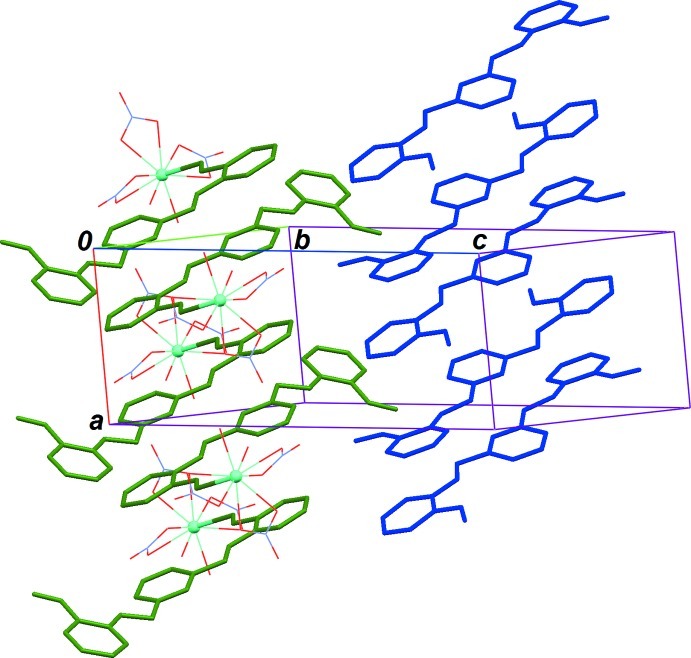
A part of the crystal structure, showing how L ligands interact in the crystal. Free L molecules (blue) form stacks separated from coordinated L molecules (green). All H atoms have been omitted for clarity.

## supplementary crystallographic information

### Comment

Lanthanides (*Ln*) are well known for giving high and rather unpredictable coordination numbers, in the range 8 to 12. For example, in the case of O-donor ligands, the coordination sphere may be completed by water molecules. Such modifications are reflected in the flexible coordination geometry of these complexes, which, in turn, has consequences on the physical properties characteristics of these metals.

While working on the synthesis of an isotypic series of *Ln*^III^ complexes with photoluminescent properties (Rodríguez De Luna *et al.*, 2010), we realised that, occasionally, a by-product crystallized with the desired complex, although elemental analysis systematically matched the expected formula. The desired complex had formula [*Ln*^III^*L*_2_(NO_3_)_3_(H_2_O)_2_] where *L* is a monodentate dialdehyde ligand, 2,2'-[pyridine-2,6-diyl-bis(methyleneoxy)]dibenzaldehyde, giving a coordination number of 10 for the metal. This compound crystallizes readily in space group *C*2/*c*. The crystallographic analysis revealed that the by-product, which crystallizes in space group *P*1, is isoformular, although the coordination number is reduced to 9, because one *L* ligand, present in the asymmetric unit, is not coordinated to the metal. The resulting formula is then [*Ln*^III^*L*(NO_3_)_3_(H_2_O)_2_].*L*, which may be seen as an organic-metalorganic system.

So far, we have detected the presence of this new complex with *Ln* = Ho^III^, Tb^III^ and Dy^III^, on the basis of unit-cell parameters (Garza Rodríguez, 2010). The X-ray characterization is however complicated by the very low yield and the poor quality of single crystals we have obtained. The present report is for *Ln* = Dy^III^, which gave a suitable refinement.

The asymmetric unit contains one complex and one free ligand (Fig. 1). The Dy^III^ atom is bonded to the monodentate *L* ligand, three bidentate nitrate ions, and two water molecules, forming nine Dy—O bonds in the range 2.320 (2)–2.460 (2) Å. The resulting coordination geometry approximates a square-face-tricapped trigonal prismatic polyhedron (polyhedral symbol in the IUPAC nomenclature: *TPRS*-9; IUPAC, 2005), with distortions from the ideal *D*_3_*_h_* symmetry induced by the nitrate bite angles (Fig. 1, inset). The organic ligand is not planar, and the peripheral ring, C15···C21/O3/O4 is twisted by 39.96 (4)° from the rest of the ligand. The free ligand is more planar, and presents a conformation reminiscent of that observed in the crystal structure of pure *L* (Rodríguez De Luna *et al.*, 2010).

The crystal structure features segregated stacks for organic and metalorganic moieties (Fig. 2). The free organic molecules are stacked in the [100] direction with π···π interactions between pyridine rings in the range 3.785 (2)–4.528 (2) Å. Because of the deviation from planarity of the whole molecule, benzaldehyde rings are less engaged in π···π interactions, with centroid-to-centroid separations in the range 4.139 (3)–5.156 (3) Å. *L* ligands bonded to the metals also interact in the same direction, and the most favorable π···π separation is found at 3.781 (2) Å.

### Experimental

The title compound was obtained by mixing 2,2'-[pyridine-2,6-diyl-bis(methyleneoxy)]dibenzaldehyde (*L*, 50 mg in 15 ml of acetonitrile) and Dy(NO_3_)_3_.5H_2_O (100 mg in 2 ml of acetonitrile), at room temperature. The mixture was refluxed for 5 h and then cooled to room temperature. After evaporation of the solvent, a few crystals of the complex were collected.

### Refinement

C-bound H atoms were placed in idealized positions, with C—H bond lengths fixed to 0.95 (aromatic CH) or 0.99 Å (methylene CH_2_). In the case of coordinated water molecules, H atoms were clearly detected in a difference map, and refined freely. Final O—H bond lengths span the range 0.71 (3)–0.86 (3) Å. Isotropic displacement parameters for H atoms were calculated as *U*_iso_(H) = 1.2*U*_eq_(carrier atom).

### Crystal data

**Table:**

[Dy(NO_3_)_3_(C_21_H_17_NO_4_)(H_2_O)_2_]·C_21_H_17_NO_4_	*Z* = 2
*M**_r_* = 1079.27	*F*(000) = 1086
Triclinic, *P*1	*D*_x_ = 1.677 Mg m^−^^3^
Hall symbol: -P 1	Mo *K*α radiation, λ = 0.71073 Å
*a* = 7.7552 (3) Å	Cell parameters from 6816 reflections
*b* = 16.1249 (8) Å	θ = 3.4–26.0°
*c* = 17.7178 (7) Å	µ = 1.84 mm^−^^1^
α = 75.531 (4)°	*T* = 136 K
β = 85.173 (3)°	Irregular, colourless
γ = 88.398 (4)°	0.43 × 0.26 × 0.12 mm
*V* = 2137.71 (16) Å^3^	

### Data collection

**Table:**

Agilent Xcalibur Atlas Gemini diffractometer	8436 independent reflections
Radiation source: Enhance (Mo) X-ray Source	7464 reflections with *I* > 2σ(*I*)
Graphite monochromator	*R*_int_ = 0.034
Detector resolution: 10.4685 pixels mm^-1^	θ_max_ = 26.1°, θ_min_ = 3.4°
φ and ω scans	*h* = −9→9
Absorption correction: analytical [*CrysAlis PRO* (Agilent, 2010); based on expressions derived by Clark & Reid (1995)]	*k* = −19→19
*T*_min_ = 0.599, *T*_max_ = 0.816	*l* = −20→21
15907 measured reflections	

### Refinement

**Table:**

Refinement on *F*^2^	Primary atom site location: structure-invariant direct methods
Least-squares matrix: full	Secondary atom site location: difference Fourier map
*R*[*F*^2^ > 2σ(*F*^2^)] = 0.030	Hydrogen site location: inferred from neighbouring sites
*wR*(*F*^2^) = 0.066	H atoms treated by a mixture of independent and constrained refinement
*S* = 1.05	*w* = 1/[σ^2^(*F*_o_^2^) + (0.021*P*)^2^ + 0.3171*P*] where *P* = (*F*_o_^2^ + 2*F*_c_^2^)/3
8436 reflections	(Δ/σ)_max_ = 0.001
616 parameters	Δρ_max_ = 0.95 e Å^−^^3^
0 restraints	Δρ_min_ = −0.88 e Å^−^^3^
0 constraints	

### Fractional atomic coordinates and isotropic or equivalent isotropic displacement parameters (Å^2^)

**Table:**

	*x*	*y*	*z*	*U*_iso_*/*U*_eq_	
Dy1	0.314191 (17)	0.208885 (9)	0.209569 (8)	0.01740 (6)	
O1	0.3943 (3)	0.27312 (13)	0.07188 (12)	0.0234 (5)	
O2	0.2481 (3)	0.50508 (13)	−0.02862 (12)	0.0256 (5)	
O3	−0.1020 (3)	0.86057 (13)	0.08702 (12)	0.0264 (5)	
O4	0.0259 (3)	0.97127 (14)	0.24948 (13)	0.0313 (5)	
N1	0.0247 (3)	0.69668 (15)	−0.00696 (14)	0.0161 (5)	
C1	0.3563 (4)	0.3456 (2)	0.03692 (18)	0.0211 (7)	
H1A	0.2981	0.3812	0.0665	0.025*	
C2	0.3930 (4)	0.38191 (18)	−0.04627 (17)	0.0165 (6)	
C3	0.4822 (4)	0.3355 (2)	−0.09437 (18)	0.0207 (7)	
H3A	0.5236	0.2795	−0.0722	0.025*	
C4	0.5106 (4)	0.3703 (2)	−0.17365 (19)	0.0259 (7)	
H4A	0.5723	0.3388	−0.2060	0.031*	
C5	0.4485 (4)	0.4517 (2)	−0.20566 (19)	0.0279 (8)	
H5A	0.4674	0.4754	−0.2604	0.033*	
C6	0.3594 (4)	0.4993 (2)	−0.15972 (19)	0.0245 (7)	
H6A	0.3174	0.5551	−0.1826	0.029*	
C7	0.3324 (4)	0.46446 (19)	−0.07995 (18)	0.0194 (7)	
C8	0.1760 (4)	0.58838 (18)	−0.05739 (18)	0.0210 (7)	
H8A	0.0827	0.5856	−0.0915	0.025*	
H8B	0.2663	0.6278	−0.0883	0.025*	
C9	0.1047 (4)	0.61991 (18)	0.01229 (17)	0.0164 (6)	
C10	0.1242 (4)	0.57589 (19)	0.08854 (18)	0.0208 (7)	
H10A	0.1813	0.5218	0.1000	0.025*	
C11	0.0588 (4)	0.6122 (2)	0.14780 (18)	0.0240 (7)	
H11A	0.0693	0.5830	0.2009	0.029*	
C12	−0.0220 (4)	0.6911 (2)	0.12971 (18)	0.0221 (7)	
H12A	−0.0668	0.7171	0.1699	0.027*	
C13	−0.0365 (4)	0.73145 (18)	0.05210 (17)	0.0175 (6)	
C14	−0.1237 (4)	0.81730 (18)	0.02744 (17)	0.0185 (6)	
H14A	−0.0699	0.8503	−0.0235	0.022*	
H14B	−0.2482	0.8101	0.0222	0.022*	
C15	−0.1588 (4)	0.94333 (19)	0.07654 (18)	0.0213 (7)	
C16	−0.2499 (4)	0.9860 (2)	0.01378 (19)	0.0230 (7)	
H16A	−0.2743	0.9586	−0.0257	0.028*	
C17	−0.3046 (4)	1.0697 (2)	0.0099 (2)	0.0261 (7)	
H17A	−0.3662	1.0995	−0.0330	0.031*	
C18	−0.2714 (4)	1.1104 (2)	0.0669 (2)	0.0291 (8)	
H18A	−0.3119	1.1672	0.0639	0.035*	
C19	−0.1788 (4)	1.06780 (19)	0.12838 (19)	0.0245 (7)	
H19A	−0.1550	1.0959	0.1675	0.029*	
C20	−0.1196 (4)	0.98422 (19)	0.13412 (18)	0.0202 (7)	
C21	−0.0134 (4)	0.9414 (2)	0.19675 (19)	0.0261 (7)	
H21A	0.0286	0.8855	0.1963	0.031*	
O5	0.0475 (3)	0.24740 (15)	0.15929 (14)	0.0233 (5)	
H51	0.026 (4)	0.259 (2)	0.117 (2)	0.028*	
H52	−0.029 (4)	0.244 (2)	0.187 (2)	0.028*	
O6	0.1312 (3)	0.11311 (14)	0.29763 (13)	0.0206 (5)	
H61	0.092 (5)	0.079 (2)	0.286 (2)	0.025*	
H62	0.150 (4)	0.097 (2)	0.346 (2)	0.025*	
N2	0.2136 (4)	0.36004 (18)	0.25775 (16)	0.0277 (6)	
N3	0.6425 (3)	0.22608 (17)	0.26502 (16)	0.0239 (6)	
N4	0.3919 (3)	0.04582 (16)	0.17732 (15)	0.0228 (6)	
O7	0.3260 (3)	0.36027 (13)	0.20041 (13)	0.0285 (5)	
O8	0.1607 (3)	0.28687 (13)	0.29638 (12)	0.0251 (5)	
O9	0.1582 (4)	0.42612 (15)	0.27163 (16)	0.0473 (7)	
O10	0.6228 (3)	0.24169 (14)	0.19230 (12)	0.0252 (5)	
O11	0.5087 (3)	0.20311 (14)	0.31135 (12)	0.0239 (5)	
O12	0.7836 (3)	0.23299 (18)	0.28843 (15)	0.0424 (7)	
O13	0.4666 (3)	0.07017 (13)	0.22969 (12)	0.0250 (5)	
O14	0.2866 (3)	0.10068 (13)	0.13961 (12)	0.0242 (5)	
O15	0.4164 (3)	−0.02375 (13)	0.16444 (13)	0.0301 (5)	
O51	0.6068 (4)	0.64204 (18)	0.57161 (17)	0.0580 (8)	
O52	0.4043 (3)	0.85788 (14)	0.45072 (13)	0.0301 (5)	
O53	−0.0234 (3)	1.21517 (14)	0.53723 (13)	0.0331 (6)	
O54	−0.1747 (3)	1.27365 (19)	0.73601 (16)	0.0518 (8)	
N51	0.1880 (3)	1.05765 (15)	0.45113 (14)	0.0179 (5)	
C51	0.5413 (5)	0.7050 (2)	0.5331 (2)	0.0361 (9)	
H51A	0.4657	0.7376	0.5597	0.043*	
C52	0.5697 (4)	0.7354 (2)	0.4473 (2)	0.0274 (8)	
C53	0.6692 (5)	0.6861 (2)	0.4054 (2)	0.0374 (9)	
H53A	0.7203	0.6341	0.4326	0.045*	
C54	0.6948 (5)	0.7116 (3)	0.3246 (2)	0.0444 (10)	
H54A	0.7638	0.6778	0.2966	0.053*	
C55	0.6194 (5)	0.7863 (2)	0.2855 (2)	0.0365 (9)	
H55A	0.6358	0.8036	0.2300	0.044*	
C56	0.5196 (4)	0.8369 (2)	0.3254 (2)	0.0285 (8)	
H56A	0.4681	0.8885	0.2976	0.034*	
C57	0.4961 (4)	0.8113 (2)	0.40600 (19)	0.0239 (7)	
C58	0.3267 (4)	0.93714 (19)	0.41339 (18)	0.0219 (7)	
H58A	0.2264	0.9267	0.3861	0.026*	
H58B	0.4114	0.9726	0.3745	0.026*	
C59	0.2691 (4)	0.98224 (19)	0.47611 (17)	0.0182 (6)	
C60	0.2996 (4)	0.9489 (2)	0.55383 (18)	0.0223 (7)	
H60A	0.3567	0.8952	0.5699	0.027*	
C61	0.2456 (4)	0.9951 (2)	0.60731 (18)	0.0247 (7)	
H61A	0.2641	0.9734	0.6610	0.030*	
C62	0.1645 (4)	1.0730 (2)	0.58215 (18)	0.0226 (7)	
H62A	0.1284	1.1062	0.6181	0.027*	
C63	0.1363 (4)	1.10250 (19)	0.50375 (17)	0.0178 (6)	
C64	0.0520 (4)	1.18726 (19)	0.47187 (18)	0.0226 (7)	
H64A	0.1388	1.2290	0.4415	0.027*	
H64B	−0.0379	1.1812	0.4372	0.027*	
C65	−0.0895 (4)	1.2957 (2)	0.5271 (2)	0.0305 (8)	
C66	−0.1002 (6)	1.3522 (2)	0.4560 (2)	0.0495 (11)	
H66A	−0.0590	1.3371	0.4092	0.059*	
C67	−0.1727 (9)	1.4322 (3)	0.4539 (3)	0.099 (2)	
H67A	−0.1809	1.4725	0.4050	0.118*	
C68	−0.2332 (9)	1.4540 (3)	0.5219 (4)	0.105 (2)	
H68A	−0.2861	1.5084	0.5191	0.126*	
C69	−0.2182 (6)	1.3984 (3)	0.5933 (3)	0.0601 (13)	
H69A	−0.2561	1.4151	0.6398	0.072*	
C70	−0.1479 (4)	1.3182 (2)	0.5976 (2)	0.0332 (8)	
C71	−0.1374 (4)	1.2578 (3)	0.6730 (2)	0.0374 (9)	
H71A	−0.0982	1.2014	0.6732	0.045*	

### Atomic displacement parameters (Å^2^)

**Table:**

	*U*^11^	*U*^22^	*U*^33^	*U*^12^	*U*^13^	*U*^23^
Dy1	0.01950 (8)	0.01749 (9)	0.01499 (8)	0.00244 (6)	−0.00209 (6)	−0.00368 (6)
O1	0.0267 (11)	0.0203 (12)	0.0201 (12)	0.0047 (10)	−0.0035 (10)	0.0006 (9)
O2	0.0428 (13)	0.0166 (11)	0.0159 (11)	0.0112 (10)	0.0027 (10)	−0.0040 (9)
O3	0.0444 (14)	0.0177 (12)	0.0212 (12)	0.0081 (10)	−0.0105 (10)	−0.0107 (10)
O4	0.0441 (14)	0.0335 (14)	0.0193 (12)	−0.0114 (11)	0.0020 (11)	−0.0122 (10)
N1	0.0185 (12)	0.0154 (13)	0.0158 (13)	0.0002 (10)	−0.0013 (10)	−0.0062 (11)
C1	0.0221 (16)	0.0213 (17)	0.0204 (17)	0.0013 (13)	0.0009 (13)	−0.0069 (14)
C2	0.0163 (14)	0.0161 (15)	0.0161 (16)	−0.0004 (12)	0.0028 (12)	−0.0036 (12)
C3	0.0202 (15)	0.0203 (16)	0.0231 (17)	0.0006 (13)	0.0013 (13)	−0.0089 (14)
C4	0.0267 (17)	0.0289 (19)	0.0245 (18)	0.0006 (15)	0.0077 (14)	−0.0145 (15)
C5	0.0331 (18)	0.033 (2)	0.0169 (17)	−0.0031 (15)	0.0046 (14)	−0.0064 (15)
C6	0.0295 (17)	0.0226 (17)	0.0194 (17)	0.0033 (14)	0.0037 (14)	−0.0035 (14)
C7	0.0221 (15)	0.0179 (16)	0.0193 (16)	−0.0020 (13)	0.0011 (13)	−0.0070 (13)
C8	0.0279 (16)	0.0150 (16)	0.0186 (16)	0.0029 (13)	−0.0007 (13)	−0.0022 (13)
C9	0.0179 (14)	0.0174 (16)	0.0152 (15)	−0.0031 (12)	0.0012 (12)	−0.0070 (12)
C10	0.0276 (16)	0.0144 (16)	0.0207 (17)	0.0021 (13)	−0.0061 (14)	−0.0040 (13)
C11	0.0337 (18)	0.0238 (18)	0.0160 (16)	0.0006 (15)	−0.0061 (14)	−0.0063 (14)
C12	0.0277 (16)	0.0223 (17)	0.0190 (17)	0.0022 (14)	−0.0005 (14)	−0.0107 (14)
C13	0.0182 (15)	0.0184 (16)	0.0176 (16)	−0.0016 (12)	−0.0035 (13)	−0.0070 (13)
C14	0.0244 (16)	0.0187 (16)	0.0151 (15)	0.0039 (13)	−0.0039 (13)	−0.0089 (13)
C15	0.0246 (16)	0.0170 (16)	0.0222 (17)	0.0002 (13)	0.0055 (14)	−0.0074 (13)
C16	0.0232 (16)	0.0235 (17)	0.0230 (17)	0.0008 (14)	−0.0011 (14)	−0.0076 (14)
C17	0.0236 (16)	0.0246 (18)	0.0294 (19)	0.0028 (14)	0.0017 (15)	−0.0067 (15)
C18	0.0270 (17)	0.0197 (17)	0.041 (2)	0.0015 (14)	0.0062 (16)	−0.0111 (16)
C19	0.0246 (16)	0.0228 (17)	0.0286 (19)	−0.0055 (14)	0.0079 (14)	−0.0136 (15)
C20	0.0231 (15)	0.0201 (16)	0.0177 (16)	−0.0036 (13)	0.0063 (13)	−0.0078 (13)
C21	0.0332 (18)	0.0243 (18)	0.0219 (18)	−0.0039 (15)	0.0044 (15)	−0.0093 (15)
O5	0.0219 (12)	0.0332 (13)	0.0132 (12)	0.0063 (10)	−0.0001 (10)	−0.0037 (11)
O6	0.0272 (12)	0.0218 (13)	0.0137 (11)	−0.0034 (10)	−0.0024 (10)	−0.0053 (10)
N2	0.0370 (16)	0.0272 (17)	0.0196 (15)	0.0022 (13)	−0.0014 (13)	−0.0077 (13)
N3	0.0205 (14)	0.0262 (15)	0.0253 (16)	0.0043 (12)	−0.0045 (12)	−0.0062 (12)
N4	0.0302 (15)	0.0198 (15)	0.0170 (14)	0.0022 (12)	0.0039 (12)	−0.0045 (12)
O7	0.0399 (13)	0.0237 (12)	0.0209 (12)	−0.0045 (10)	0.0055 (11)	−0.0060 (10)
O8	0.0316 (12)	0.0209 (12)	0.0205 (12)	−0.0005 (10)	−0.0007 (10)	−0.0011 (10)
O9	0.077 (2)	0.0240 (14)	0.0404 (16)	0.0164 (14)	0.0043 (14)	−0.0112 (12)
O10	0.0271 (12)	0.0312 (13)	0.0158 (12)	−0.0012 (10)	−0.0016 (9)	−0.0029 (10)
O11	0.0202 (11)	0.0332 (13)	0.0176 (12)	0.0012 (10)	−0.0009 (9)	−0.0055 (10)
O12	0.0208 (12)	0.0715 (19)	0.0357 (15)	−0.0012 (12)	−0.0079 (11)	−0.0128 (14)
O13	0.0272 (12)	0.0265 (12)	0.0228 (12)	0.0061 (10)	−0.0047 (10)	−0.0090 (10)
O14	0.0349 (12)	0.0212 (12)	0.0161 (11)	0.0071 (10)	−0.0061 (10)	−0.0033 (9)
O15	0.0445 (14)	0.0171 (12)	0.0293 (13)	0.0045 (10)	0.0012 (11)	−0.0086 (10)
O51	0.084 (2)	0.0463 (17)	0.0405 (17)	0.0355 (17)	−0.0179 (16)	−0.0053 (14)
O52	0.0435 (14)	0.0244 (12)	0.0208 (12)	0.0154 (11)	−0.0006 (11)	−0.0049 (10)
O53	0.0537 (15)	0.0250 (13)	0.0215 (13)	0.0138 (11)	0.0022 (11)	−0.0104 (10)
O54	0.0451 (16)	0.084 (2)	0.0402 (17)	0.0168 (15)	−0.0043 (13)	−0.0427 (16)
N51	0.0216 (13)	0.0179 (13)	0.0140 (13)	−0.0004 (11)	−0.0001 (11)	−0.0041 (11)
C51	0.049 (2)	0.031 (2)	0.029 (2)	0.0160 (18)	−0.0084 (18)	−0.0084 (17)
C52	0.0276 (17)	0.0278 (19)	0.0288 (19)	0.0035 (15)	−0.0062 (15)	−0.0099 (15)
C53	0.041 (2)	0.030 (2)	0.042 (2)	0.0139 (17)	−0.0028 (18)	−0.0109 (18)
C54	0.049 (2)	0.042 (2)	0.045 (3)	0.0113 (19)	0.010 (2)	−0.021 (2)
C55	0.043 (2)	0.040 (2)	0.028 (2)	−0.0011 (18)	0.0044 (17)	−0.0139 (17)
C56	0.0352 (19)	0.0259 (18)	0.0248 (19)	0.0005 (15)	0.0009 (15)	−0.0080 (15)
C57	0.0238 (16)	0.0230 (17)	0.0273 (18)	0.0005 (14)	−0.0008 (14)	−0.0112 (15)
C58	0.0262 (16)	0.0202 (17)	0.0186 (17)	0.0056 (14)	−0.0034 (13)	−0.0038 (13)
C59	0.0171 (15)	0.0211 (17)	0.0166 (16)	−0.0021 (13)	0.0015 (13)	−0.0056 (13)
C60	0.0228 (16)	0.0233 (17)	0.0188 (17)	0.0028 (13)	−0.0043 (13)	−0.0013 (14)
C61	0.0249 (16)	0.0321 (19)	0.0153 (16)	0.0004 (15)	−0.0017 (14)	−0.0025 (14)
C62	0.0231 (16)	0.0274 (18)	0.0182 (17)	−0.0026 (14)	−0.0001 (13)	−0.0078 (14)
C63	0.0171 (14)	0.0203 (16)	0.0167 (16)	−0.0035 (13)	0.0006 (12)	−0.0063 (13)
C64	0.0319 (17)	0.0218 (17)	0.0151 (16)	0.0007 (14)	0.0007 (14)	−0.0073 (13)
C65	0.0281 (18)	0.0248 (19)	0.039 (2)	0.0023 (15)	−0.0006 (16)	−0.0104 (16)
C66	0.071 (3)	0.031 (2)	0.041 (3)	0.017 (2)	0.007 (2)	−0.0037 (19)
C67	0.159 (6)	0.047 (3)	0.064 (4)	0.048 (4)	0.035 (4)	0.013 (3)
C68	0.167 (6)	0.036 (3)	0.093 (5)	0.048 (3)	0.044 (4)	−0.004 (3)
C69	0.072 (3)	0.037 (2)	0.070 (3)	0.009 (2)	0.024 (3)	−0.022 (2)
C70	0.0275 (18)	0.030 (2)	0.048 (2)	0.0031 (15)	−0.0002 (17)	−0.0211 (18)
C71	0.0324 (19)	0.051 (2)	0.037 (2)	0.0130 (18)	−0.0060 (17)	−0.027 (2)

### Geometric parameters (Å, º)

**Table:**

Dy1—O1	2.435 (2)	O5—H52	0.73 (3)
Dy1—O5	2.327 (2)	O6—H61	0.71 (3)
Dy1—O6	2.320 (2)	O6—H62	0.86 (3)
Dy1—O7	2.410 (2)	N2—O9	1.212 (3)
Dy1—O8	2.437 (2)	N2—O8	1.267 (3)
Dy1—O10	2.443 (2)	N2—O7	1.281 (3)
Dy1—O11	2.429 (2)	N3—O12	1.221 (3)
Dy1—O13	2.460 (2)	N3—O11	1.270 (3)
Dy1—O14	2.403 (2)	N3—O10	1.271 (3)
Dy1—N2	2.846 (3)	N4—O15	1.206 (3)
Dy1—N3	2.849 (3)	N4—O13	1.279 (3)
Dy1—N4	2.865 (3)	N4—O14	1.285 (3)
O1—C1	1.220 (3)	O51—C51	1.199 (4)
O2—C7	1.363 (3)	O52—C57	1.368 (4)
O2—C8	1.428 (3)	O52—C58	1.426 (3)
O3—C15	1.366 (3)	O53—C65	1.358 (4)
O3—C14	1.426 (3)	O53—C64	1.420 (3)
O4—C21	1.216 (4)	O54—C71	1.217 (4)
N1—C9	1.347 (4)	N51—C59	1.344 (4)
N1—C13	1.353 (4)	N51—C63	1.346 (4)
C1—C2	1.450 (4)	C51—C52	1.475 (5)
C1—H1A	0.9500	C51—H51A	0.9500
C2—C3	1.398 (4)	C52—C57	1.394 (4)
C2—C7	1.402 (4)	C52—C53	1.395 (5)
C3—C4	1.377 (4)	C53—C54	1.386 (5)
C3—H3A	0.9500	C53—H53A	0.9500
C4—C5	1.384 (4)	C54—C55	1.373 (5)
C4—H4A	0.9500	C54—H54A	0.9500
C5—C6	1.385 (4)	C55—C56	1.388 (5)
C5—H5A	0.9500	C55—H55A	0.9500
C6—C7	1.385 (4)	C56—C57	1.382 (5)
C6—H6A	0.9500	C56—H56A	0.9500
C8—C9	1.507 (4)	C58—C59	1.505 (4)
C8—H8A	0.9900	C58—H58A	0.9900
C8—H8B	0.9900	C58—H58B	0.9900
C9—C10	1.379 (4)	C59—C60	1.385 (4)
C10—C11	1.380 (4)	C60—C61	1.377 (4)
C10—H10A	0.9500	C60—H60A	0.9500
C11—C12	1.378 (4)	C61—C62	1.378 (4)
C11—H11A	0.9500	C61—H61A	0.9500
C12—C13	1.379 (4)	C62—C63	1.385 (4)
C12—H12A	0.9500	C62—H62A	0.9500
C13—C14	1.503 (4)	C63—C64	1.497 (4)
C14—H14A	0.9900	C64—H64A	0.9900
C14—H14B	0.9900	C64—H64B	0.9900
C15—C16	1.390 (4)	C65—C66	1.366 (5)
C15—C20	1.403 (4)	C65—C70	1.419 (5)
C16—C17	1.389 (4)	C66—C67	1.385 (6)
C16—H16A	0.9500	C66—H66A	0.9500
C17—C18	1.379 (5)	C67—C68	1.380 (7)
C17—H17A	0.9500	C67—H67A	0.9500
C18—C19	1.380 (5)	C68—C69	1.366 (7)
C18—H18A	0.9500	C68—H68A	0.9500
C19—C20	1.394 (4)	C69—C70	1.377 (5)
C19—H19A	0.9500	C69—H69A	0.9500
C20—C21	1.458 (4)	C70—C71	1.451 (5)
C21—H21A	0.9500	C71—H71A	0.9500
O5—H51	0.76 (4)		
			
O6—Dy1—O5	78.55 (8)	C17—C16—C15	118.7 (3)
O6—Dy1—O14	79.03 (8)	C17—C16—H16A	120.6
O5—Dy1—O14	79.82 (8)	C15—C16—H16A	120.6
O6—Dy1—O7	125.04 (7)	C18—C17—C16	121.4 (3)
O5—Dy1—O7	81.96 (8)	C18—C17—H17A	119.3
O14—Dy1—O7	145.81 (7)	C16—C17—H17A	119.3
O6—Dy1—O11	89.94 (7)	C17—C18—C19	119.4 (3)
O5—Dy1—O11	149.62 (8)	C17—C18—H18A	120.3
O14—Dy1—O11	125.81 (7)	C19—C18—H18A	120.3
O7—Dy1—O11	81.97 (7)	C18—C19—C20	121.1 (3)
O6—Dy1—O1	145.20 (8)	C18—C19—H19A	119.4
O5—Dy1—O1	77.24 (8)	C20—C19—H19A	119.4
O14—Dy1—O1	72.40 (7)	C19—C20—C15	118.5 (3)
O7—Dy1—O1	75.50 (7)	C19—C20—C21	120.8 (3)
O11—Dy1—O1	122.85 (7)	C15—C20—C21	120.6 (3)
O6—Dy1—O8	72.43 (7)	O4—C21—C20	125.3 (3)
O5—Dy1—O8	74.04 (8)	O4—C21—H21A	117.4
O14—Dy1—O8	144.36 (7)	C20—C21—H21A	117.4
O7—Dy1—O8	52.83 (7)	Dy1—O5—H51	129 (3)
O11—Dy1—O8	75.70 (7)	Dy1—O5—H52	118 (3)
O1—Dy1—O8	123.16 (7)	H51—O5—H52	113 (4)
O6—Dy1—O10	136.95 (7)	Dy1—O6—H61	121 (3)
O5—Dy1—O10	144.49 (8)	Dy1—O6—H62	121 (2)
O14—Dy1—O10	104.30 (7)	H61—O6—H62	110 (4)
O7—Dy1—O10	75.42 (7)	O9—N2—O8	122.9 (3)
O11—Dy1—O10	52.72 (7)	O9—N2—O7	121.5 (3)
O1—Dy1—O10	70.85 (7)	O8—N2—O7	115.6 (2)
O8—Dy1—O10	111.08 (7)	O9—N2—Dy1	172.6 (2)
O6—Dy1—O13	75.04 (7)	O8—N2—Dy1	58.53 (14)
O5—Dy1—O13	128.94 (8)	O7—N2—Dy1	57.34 (14)
O14—Dy1—O13	52.83 (7)	O12—N3—O11	122.2 (3)
O7—Dy1—O13	148.23 (7)	O12—N3—O10	121.0 (3)
O11—Dy1—O13	73.02 (7)	O11—N3—O10	116.7 (2)
O1—Dy1—O13	101.80 (7)	O12—N3—Dy1	179.5 (2)
O8—Dy1—O13	134.29 (7)	O11—N3—Dy1	58.04 (14)
O10—Dy1—O13	73.92 (7)	O10—N3—Dy1	58.66 (14)
O6—Dy1—N2	98.47 (8)	O15—N4—O13	122.9 (3)
O5—Dy1—N2	75.03 (8)	O15—N4—O14	122.1 (3)
O14—Dy1—N2	154.70 (7)	O13—N4—O14	115.1 (2)
O7—Dy1—N2	26.59 (7)	O15—N4—Dy1	176.9 (2)
O11—Dy1—N2	79.08 (7)	O13—N4—Dy1	58.85 (13)
O1—Dy1—N2	98.99 (7)	O14—N4—Dy1	56.29 (13)
O8—Dy1—N2	26.32 (7)	N2—O7—Dy1	96.07 (17)
O10—Dy1—N2	94.69 (8)	N2—O8—Dy1	95.15 (17)
O13—Dy1—N2	151.25 (7)	N3—O10—Dy1	94.95 (17)
O6—Dy1—N3	113.96 (8)	N3—O11—Dy1	95.62 (16)
O5—Dy1—N3	159.45 (8)	N4—O13—Dy1	94.73 (16)
O14—Dy1—N3	117.58 (7)	N4—O14—Dy1	97.28 (16)
O7—Dy1—N3	77.50 (7)	C57—O52—C58	119.3 (2)
O11—Dy1—N3	26.34 (7)	C65—O53—C64	120.0 (3)
O1—Dy1—N3	96.91 (7)	C59—N51—C63	118.5 (2)
O8—Dy1—N3	93.67 (7)	O51—C51—C52	124.7 (3)
O10—Dy1—N3	26.38 (7)	O51—C51—H51A	117.7
O13—Dy1—N3	71.37 (7)	C52—C51—H51A	117.7
N2—Dy1—N3	86.66 (8)	C57—C52—C53	118.4 (3)
O6—Dy1—N4	74.87 (7)	C57—C52—C51	122.1 (3)
O5—Dy1—N4	104.39 (8)	C53—C52—C51	119.5 (3)
O14—Dy1—N4	26.42 (7)	C54—C53—C52	121.0 (3)
O7—Dy1—N4	160.10 (7)	C54—C53—H53A	119.5
O11—Dy1—N4	99.44 (7)	C52—C53—H53A	119.5
O1—Dy1—N4	87.39 (7)	C55—C54—C53	119.2 (3)
O8—Dy1—N4	146.90 (7)	C55—C54—H54A	120.4
O10—Dy1—N4	89.55 (7)	C53—C54—H54A	120.4
O13—Dy1—N4	26.42 (7)	C54—C55—C56	121.2 (3)
N2—Dy1—N4	173.22 (7)	C54—C55—H55A	119.4
N3—Dy1—N4	94.89 (7)	C56—C55—H55A	119.4
C1—O1—Dy1	125.07 (19)	C57—C56—C55	119.1 (3)
C7—O2—C8	119.3 (2)	C57—C56—H56A	120.4
C15—O3—C14	118.7 (2)	C55—C56—H56A	120.4
C9—N1—C13	117.5 (2)	O52—C57—C56	123.6 (3)
O1—C1—C2	125.1 (3)	O52—C57—C52	115.5 (3)
O1—C1—H1A	117.4	C56—C57—C52	121.0 (3)
C2—C1—H1A	117.4	O52—C58—C59	107.3 (2)
C3—C2—C7	119.0 (3)	O52—C58—H58A	110.3
C3—C2—C1	121.7 (3)	C59—C58—H58A	110.3
C7—C2—C1	119.3 (3)	O52—C58—H58B	110.3
C4—C3—C2	120.7 (3)	C59—C58—H58B	110.3
C4—C3—H3A	119.7	H58A—C58—H58B	108.5
C2—C3—H3A	119.7	N51—C59—C60	122.5 (3)
C3—C4—C5	119.3 (3)	N51—C59—C58	115.2 (2)
C3—C4—H4A	120.3	C60—C59—C58	122.4 (3)
C5—C4—H4A	120.3	C61—C60—C59	118.7 (3)
C4—C5—C6	121.5 (3)	C61—C60—H60A	120.7
C4—C5—H5A	119.2	C59—C60—H60A	120.7
C6—C5—H5A	119.2	C60—C61—C62	119.4 (3)
C7—C6—C5	119.0 (3)	C60—C61—H61A	120.3
C7—C6—H6A	120.5	C62—C61—H61A	120.3
C5—C6—H6A	120.5	C61—C62—C63	119.2 (3)
O2—C7—C6	124.6 (3)	C61—C62—H62A	120.4
O2—C7—C2	114.9 (3)	C63—C62—H62A	120.4
C6—C7—C2	120.5 (3)	N51—C63—C62	121.9 (3)
O2—C8—C9	107.6 (2)	N51—C63—C64	115.9 (3)
O2—C8—H8A	110.2	C62—C63—C64	122.2 (3)
C9—C8—H8A	110.2	O53—C64—C63	106.6 (2)
O2—C8—H8B	110.2	O53—C64—H64A	110.4
C9—C8—H8B	110.2	C63—C64—H64A	110.4
H8A—C8—H8B	108.5	O53—C64—H64B	110.4
N1—C9—C10	123.0 (3)	C63—C64—H64B	110.4
N1—C9—C8	113.6 (2)	H64A—C64—H64B	108.6
C10—C9—C8	123.3 (3)	O53—C65—C66	124.2 (3)
C9—C10—C11	118.4 (3)	O53—C65—C70	114.5 (3)
C9—C10—H10A	120.8	C66—C65—C70	121.3 (3)
C11—C10—H10A	120.8	C65—C66—C67	118.3 (4)
C12—C11—C10	119.7 (3)	C65—C66—H66A	120.9
C12—C11—H11A	120.1	C67—C66—H66A	120.9
C10—C11—H11A	120.1	C68—C67—C66	120.8 (5)
C11—C12—C13	118.6 (3)	C68—C67—H67A	119.6
C11—C12—H12A	120.7	C66—C67—H67A	119.6
C13—C12—H12A	120.7	C69—C68—C67	121.0 (4)
N1—C13—C12	122.7 (3)	C69—C68—H68A	119.5
N1—C13—C14	115.3 (2)	C67—C68—H68A	119.5
C12—C13—C14	121.9 (3)	C68—C69—C70	119.7 (4)
O3—C14—C13	106.5 (2)	C68—C69—H69A	120.1
O3—C14—H14A	110.4	C70—C69—H69A	120.1
C13—C14—H14A	110.4	C69—C70—C65	118.8 (4)
O3—C14—H14B	110.4	C69—C70—C71	120.0 (4)
C13—C14—H14B	110.4	C65—C70—C71	121.2 (3)
H14A—C14—H14B	108.6	O54—C71—C70	125.2 (3)
O3—C15—C16	123.6 (3)	O54—C71—H71A	117.4
O3—C15—C20	115.6 (3)	C70—C71—H71A	117.4
C16—C15—C20	120.8 (3)		
			
O6—Dy1—O1—C1	104.2 (2)	O6—Dy1—O7—N2	2.6 (2)
O5—Dy1—O1—C1	57.2 (2)	O5—Dy1—O7—N2	72.50 (17)
O14—Dy1—O1—C1	140.4 (2)	O14—Dy1—O7—N2	130.81 (17)
O7—Dy1—O1—C1	−27.7 (2)	O11—Dy1—O7—N2	−81.64 (17)
O11—Dy1—O1—C1	−97.9 (2)	O1—Dy1—O7—N2	151.32 (18)
O8—Dy1—O1—C1	−3.8 (3)	O8—Dy1—O7—N2	−3.53 (15)
O10—Dy1—O1—C1	−107.0 (2)	O10—Dy1—O7—N2	−135.10 (18)
O13—Dy1—O1—C1	−175.1 (2)	O13—Dy1—O7—N2	−119.63 (18)
N2—Dy1—O1—C1	−15.1 (2)	N3—Dy1—O7—N2	−108.03 (17)
N3—Dy1—O1—C1	−102.8 (2)	N4—Dy1—O7—N2	−177.18 (19)
N4—Dy1—O1—C1	162.6 (2)	O9—N2—O8—Dy1	171.4 (3)
Dy1—O1—C1—C2	−175.3 (2)	O7—N2—O8—Dy1	−5.9 (3)
O1—C1—C2—C3	−1.7 (5)	O6—Dy1—O8—N2	−171.17 (18)
O1—C1—C2—C7	176.0 (3)	O5—Dy1—O8—N2	−88.41 (18)
C7—C2—C3—C4	0.2 (4)	O14—Dy1—O8—N2	−132.82 (17)
C1—C2—C3—C4	177.9 (3)	O7—Dy1—O8—N2	3.56 (16)
C2—C3—C4—C5	−0.6 (5)	O11—Dy1—O8—N2	94.27 (17)
C3—C4—C5—C6	0.5 (5)	O1—Dy1—O8—N2	−25.88 (19)
C4—C5—C6—C7	0.1 (5)	O10—Dy1—O8—N2	54.45 (18)
C8—O2—C7—C6	2.1 (4)	O13—Dy1—O8—N2	142.22 (16)
C8—O2—C7—C2	−177.9 (2)	N3—Dy1—O8—N2	74.84 (17)
C5—C6—C7—O2	179.5 (3)	N4—Dy1—O8—N2	179.61 (15)
C5—C6—C7—C2	−0.6 (4)	O12—N3—O10—Dy1	179.5 (3)
C3—C2—C7—O2	−179.6 (3)	O11—N3—O10—Dy1	−0.4 (3)
C1—C2—C7—O2	2.7 (4)	O6—Dy1—O10—N3	−35.0 (2)
C3—C2—C7—C6	0.4 (4)	O5—Dy1—O10—N3	143.38 (17)
C1—C2—C7—C6	−177.3 (3)	O14—Dy1—O10—N3	−124.13 (16)
C7—O2—C8—C9	−175.5 (2)	O7—Dy1—O10—N3	91.21 (17)
C13—N1—C9—C10	0.8 (4)	O11—Dy1—O10—N3	0.26 (15)
C13—N1—C9—C8	−177.2 (2)	O1—Dy1—O10—N3	170.63 (18)
O2—C8—C9—N1	−176.9 (2)	O8—Dy1—O10—N3	51.50 (17)
O2—C8—C9—C10	5.1 (4)	O13—Dy1—O10—N3	−80.39 (16)
N1—C9—C10—C11	0.0 (4)	N2—Dy1—O10—N3	72.73 (17)
C8—C9—C10—C11	177.8 (3)	N4—Dy1—O10—N3	−101.98 (17)
C9—C10—C11—C12	−0.7 (4)	O12—N3—O11—Dy1	−179.5 (3)
C10—C11—C12—C13	0.6 (4)	O10—N3—O11—Dy1	0.4 (3)
C9—N1—C13—C12	−0.9 (4)	O6—Dy1—O11—N3	156.54 (16)
C9—N1—C13—C14	179.7 (2)	O5—Dy1—O11—N3	−136.68 (18)
C11—C12—C13—N1	0.2 (5)	O14—Dy1—O11—N3	80.16 (18)
C11—C12—C13—C14	179.6 (3)	O7—Dy1—O11—N3	−78.01 (16)
C15—O3—C14—C13	175.3 (2)	O1—Dy1—O11—N3	−11.10 (19)
N1—C13—C14—O3	−152.1 (2)	O8—Dy1—O11—N3	−131.59 (17)
C12—C13—C14—O3	28.4 (4)	O10—Dy1—O11—N3	−0.26 (15)
C14—O3—C15—C16	5.5 (4)	O13—Dy1—O11—N3	82.18 (16)
C14—O3—C15—C20	−174.6 (3)	N2—Dy1—O11—N3	−104.82 (17)
O3—C15—C16—C17	178.7 (3)	N4—Dy1—O11—N3	81.90 (16)
C20—C15—C16—C17	−1.3 (5)	O15—N4—O13—Dy1	−176.9 (2)
C15—C16—C17—C18	−0.4 (5)	O14—N4—O13—Dy1	2.3 (2)
C16—C17—C18—C19	1.3 (5)	O6—Dy1—O13—N4	86.02 (16)
C17—C18—C19—C20	−0.5 (5)	O5—Dy1—O13—N4	24.7 (2)
C18—C19—C20—C15	−1.1 (4)	O14—Dy1—O13—N4	−1.42 (15)
C18—C19—C20—C21	176.4 (3)	O7—Dy1—O13—N4	−139.78 (17)
O3—C15—C20—C19	−177.9 (3)	O11—Dy1—O13—N4	−179.37 (17)
C16—C15—C20—C19	2.0 (4)	O1—Dy1—O13—N4	−58.33 (17)
O3—C15—C20—C21	4.5 (4)	O8—Dy1—O13—N4	131.83 (16)
C16—C15—C20—C21	−175.5 (3)	O10—Dy1—O13—N4	−124.19 (17)
C19—C20—C21—O4	3.3 (5)	N2—Dy1—O13—N4	166.22 (16)
C15—C20—C21—O4	−179.2 (3)	N3—Dy1—O13—N4	−151.73 (17)
O6—Dy1—N2—O8	8.51 (18)	O15—N4—O14—Dy1	176.9 (2)
O5—Dy1—N2—O8	84.18 (17)	O13—N4—O14—Dy1	−2.4 (2)
O14—Dy1—N2—O8	90.7 (2)	O6—Dy1—O14—N4	−78.03 (16)
O7—Dy1—N2—O8	−173.6 (3)	O5—Dy1—O14—N4	−158.21 (17)
O11—Dy1—N2—O8	−79.78 (17)	O7—Dy1—O14—N4	142.92 (16)
O1—Dy1—N2—O8	158.29 (16)	O11—Dy1—O14—N4	3.85 (19)
O10—Dy1—N2—O8	−130.38 (17)	O1—Dy1—O14—N4	122.07 (17)
O13—Dy1—N2—O8	−65.8 (2)	O8—Dy1—O14—N4	−115.09 (17)
N3—Dy1—N2—O8	−105.23 (17)	O10—Dy1—O14—N4	57.91 (17)
O6—Dy1—N2—O7	−177.84 (17)	O13—Dy1—O14—N4	1.42 (15)
O5—Dy1—N2—O7	−102.17 (18)	N2—Dy1—O14—N4	−164.64 (17)
O14—Dy1—N2—O7	−95.6 (2)	N3—Dy1—O14—N4	33.40 (18)
O11—Dy1—N2—O7	93.87 (17)	O51—C51—C52—C57	−177.0 (4)
O1—Dy1—N2—O7	−28.06 (17)	O51—C51—C52—C53	4.8 (6)
O8—Dy1—N2—O7	173.6 (3)	C57—C52—C53—C54	0.0 (5)
O10—Dy1—N2—O7	43.27 (17)	C51—C52—C53—C54	178.3 (4)
O13—Dy1—N2—O7	107.9 (2)	C52—C53—C54—C55	−0.6 (6)
N3—Dy1—N2—O7	68.42 (17)	C53—C54—C55—C56	0.6 (6)
O6—Dy1—N3—O11	−25.82 (18)	C54—C55—C56—C57	0.0 (5)
O5—Dy1—N3—O11	98.7 (3)	C58—O52—C57—C56	0.3 (4)
O14—Dy1—N3—O11	−115.66 (16)	C58—O52—C57—C52	179.0 (3)
O7—Dy1—N3—O11	97.20 (16)	C55—C56—C57—O52	178.1 (3)
O1—Dy1—N3—O11	170.62 (16)	C55—C56—C57—C52	−0.6 (5)
O8—Dy1—N3—O11	46.57 (16)	C53—C52—C57—O52	−178.2 (3)
O10—Dy1—N3—O11	179.5 (3)	C51—C52—C57—O52	3.6 (5)
O13—Dy1—N3—O11	−89.21 (16)	C53—C52—C57—C56	0.6 (5)
N2—Dy1—N3—O11	71.96 (16)	C51—C52—C57—C56	−177.7 (3)
N4—Dy1—N3—O11	−101.42 (16)	C57—O52—C58—C59	−168.1 (3)
O6—Dy1—N3—O10	154.64 (16)	C63—N51—C59—C60	0.6 (4)
O5—Dy1—N3—O10	−80.8 (3)	C63—N51—C59—C58	−178.5 (3)
O14—Dy1—N3—O10	64.81 (18)	O52—C58—C59—N51	−178.2 (2)
O7—Dy1—N3—O10	−82.34 (17)	O52—C58—C59—C60	2.7 (4)
O11—Dy1—N3—O10	−179.5 (3)	N51—C59—C60—C61	−0.4 (5)
O1—Dy1—N3—O10	−8.91 (17)	C58—C59—C60—C61	178.7 (3)
O8—Dy1—N3—O10	−132.96 (16)	C59—C60—C61—C62	−0.5 (5)
O13—Dy1—N3—O10	91.25 (17)	C60—C61—C62—C63	1.2 (4)
N2—Dy1—N3—O10	−107.58 (17)	C59—N51—C63—C62	0.0 (4)
N4—Dy1—N3—O10	79.04 (17)	C59—N51—C63—C64	178.1 (3)
O6—Dy1—N4—O13	−86.75 (16)	C61—C62—C63—N51	−0.9 (5)
O5—Dy1—N4—O13	−160.39 (16)	C61—C62—C63—C64	−178.8 (3)
O14—Dy1—N4—O13	177.5 (3)	C65—O53—C64—C63	171.2 (3)
O7—Dy1—N4—O13	93.1 (3)	N51—C63—C64—O53	168.3 (3)
O11—Dy1—N4—O13	0.61 (17)	C62—C63—C64—O53	−13.7 (4)
O1—Dy1—N4—O13	123.49 (16)	C64—O53—C65—C66	4.7 (5)
O8—Dy1—N4—O13	−77.6 (2)	C64—O53—C65—C70	−175.0 (3)
O10—Dy1—N4—O13	52.64 (16)	O53—C65—C66—C67	179.2 (4)
N3—Dy1—N4—O13	26.77 (17)	C70—C65—C66—C67	−1.1 (7)
O6—Dy1—N4—O14	95.80 (17)	C65—C66—C67—C68	−0.3 (9)
O5—Dy1—N4—O14	22.16 (17)	C66—C67—C68—C69	2.1 (11)
O7—Dy1—N4—O14	−84.4 (3)	C67—C68—C69—C70	−2.6 (10)
O11—Dy1—N4—O14	−176.84 (15)	C68—C69—C70—C65	1.2 (7)
O1—Dy1—N4—O14	−53.96 (16)	C68—C69—C70—C71	−177.8 (5)
O8—Dy1—N4—O14	104.90 (18)	O53—C65—C70—C69	−179.6 (3)
O10—Dy1—N4—O14	−124.81 (16)	C66—C65—C70—C69	0.7 (5)
O13—Dy1—N4—O14	−177.5 (3)	O53—C65—C70—C71	−0.7 (5)
N3—Dy1—N4—O14	−150.68 (16)	C66—C65—C70—C71	179.6 (4)
O9—N2—O7—Dy1	−171.4 (3)	C69—C70—C71—O54	−5.8 (6)
O8—N2—O7—Dy1	6.0 (3)	C65—C70—C71—O54	175.3 (3)

### Hydrogen-bond geometry (Å, º)

**Table:**

*D*—H···*A*	*D*—H	H···*A*	*D*···*A*	*D*—H···*A*
O5—H51···N1^i^	0.76 (4)	1.97 (4)	2.724 (3)	173 (4)
O5—H52···O12^ii^	0.73 (3)	2.19 (4)	2.907 (3)	168 (4)
O6—H61···O4^iii^	0.71 (3)	2.10 (3)	2.797 (3)	169 (4)
O6—H62···N51^iii^	0.86 (3)	1.86 (4)	2.712 (3)	177 (3)

Symmetry codes: (i) −*x*, −*y*+1, −*z*; (ii) *x*−1, *y*, *z*; (iii) *x*, *y*−1, *z*.
